# Personal Heart Health Monitoring Based on 1D Convolutional Neural Network

**DOI:** 10.3390/jimaging7020026

**Published:** 2021-02-05

**Authors:** Antonella Nannavecchia, Francesco Girardi, Pio Raffaele Fina, Michele Scalera, Giovanni Dimauro

**Affiliations:** 1 Department of Management, Finance and Technology, University LUM Jean Monnet, 70010 Casamassima, Italy; nannavecchia@lum.it; 2 UVARP Azienda Sanitaria Locale, 70132 Bari, Italy; francesco.girardi@asl.bari.it; 3 Department of Computer Science, University of Torino, 10124 Torino, Italy; pio.fina@edu.unito.it; 4 Department of Computer Science, University of Bari, 70125 Bari, Italy; michele.scalera@uniba.it

**Keywords:** ECG signal detection, portable monitoring devices, 1D-convolutional neural network, deep learning

## Abstract

The automated detection of suspicious anomalies in electrocardiogram (ECG) recordings allows frequent personal heart health monitoring and can drastically reduce the number of ECGs that need to be manually examined by the cardiologists, excluding those classified as normal, facilitating healthcare decision-making and reducing a considerable amount of time and money. In this paper, we present a system able to automatically detect the suspect of cardiac pathologies in ECG signals from personal monitoring devices, with the aim to alert the patient to send the ECG to the medical specialist for a correct diagnosis and a proper therapy. The main contributes of this work are: (a) the implementation of a binary classifier based on a 1D-CNN architecture for detecting the suspect of anomalies in ECGs, regardless of the kind of cardiac pathology; (b) the analysis was carried out on 21 classes of different cardiac pathologies classified as anomalous; and (c) the possibility to classify anomalies even in ECG segments containing, at the same time, more than one class of cardiac pathologies. Moreover, 1D-CNN based architectures can allow an implementation of the system on cheap smart devices with low computational complexity. The system was tested on the ECG signals from the MIT-BIH ECG Arrhythmia Database for the MLII derivation. Two different experiments were carried out, showing remarkable performance compared to other similar systems. The best result showed high accuracy and recall, computed in terms of ECG segments and even higher accuracy and recall in terms of patients alerted, therefore considering the detection of anomalies with respect to entire ECG recordings.

## 1. Introduction

The aging of the population is leading to an increase in patients suffering from cardiac pathologies, therefore requiring electrocardiographic monitoring. An electrocardiogram (ECG) is an easy, rapid, and non-invasive tool that traces the electrical activity of the heart [1] revealing the presence of cardiac pathologies such as conduction disease, channelopathies, structural heart disease, and previous ischemic injury [2]. On the other hand, investigating the altered acoustic characteristics of the cardiac tones, as an example, may allow the early identification of valve malfunction [3].

Systems able to support the doctors’ work in the diagnosis of pathologies can facilitate health care decision making reducing considerably expenditure of time and money [4,5,6,7,8,9,10,11,12]. The ECG has become the diagnostic procedure most commonly performed in clinical cardiology [13,14,15] and the diffusion of wearable and portable devices has been enabling patients to constantly monitor the cardiac activity, for example of elder people through wireless sensor networks [16]. Cardiologists cannot examine millions of ECGs daily recorded from portable devices. Thus, systems able to automatically detect suspicious anomalies in ECGs are required, in order to reduce the number of ECGs that need to be manually examined by the cardiologists, identifying those that need a further examination and also the urgency of such examination. For this reason, systems require high detection performance in order to avoid that normal ECGs incorrectly detected as anomalous should be examined by a medical professional, and, even more important, that the presence of an electrocardiographic alteration, which could be the indicator of cardiac pathology, is recognized and does not escape the observation of the cardiologist. To make the anomalous ECGs be examined by the medical specialist and that the proper therapy is administered, the detection system should maximize recall for anomalous ECGs, which is to maximize the number of ECGs correctly classified as anomalous, even losing accuracy.

The future of quick and efficient disease diagnosis lays in the development of reliable non-invasive methods [17] also through the use of artificial intelligence techniques. Artificial neural networks and deep learning architectures have recently found broad application [18] achieving striking success in different domains such as image classification [19,20,21,22,23,24], speech recognition [25], intrusion detection systems [26,27], smart city [28], or biological studies [29,30].

Therefore, high expectations are placed in the use of such techniques also for the improvement of health care and clinical practice [31,32,33,34,35,36]. Furthermore, numerous portable devices for personal and frequent monitoring of cardiac activity, such as Kardia [37], D-hearth [38], and eKuore [39], are spreading.

The goal of this paper is to implement a system able to automatically detect the suspect of cardiac pathologies in ECG signals to support personal monitoring devices. We propose a 1D-CNN architecture optimized to detect anomalous ECG recordings, regardless of the kind of cardiac pathology, including in the analysis 21 classes of anomalies.

The system here presented was designed to be implemented on devices for personal use and with the aim to only send to the cardiologist ECGs detected with the suspect of a cardiac alteration for further examination, thus no information about the specific class of anomaly is detected. The proposed system is based on a binary classification model.

In fact, as of now, we want to make it clear that the main goal of our study is not to classify different cardiac pathologies, but to make sure that the suspect of a pathology can be detected and that patients are alarmed: then a correct diagnosis can be carried on with specific tests and the intervention of medical staff. The cardiologist will examine all ECGs detected as anomalous identifying the pathology and prescribing the proper treatment. The system has been implemented with the aim to achieve high levels of recall for anomalous ECGs in order to minimize the possibility that the presence of any kind of cardiac alteration could escape the observation of the cardiologist.

This paper is organized as follows: Section 2 reports a wide background, Section 3 describes ECG signals; Section 4 illustrates material and methods; in Section 5 are pointed out results and discussions; and Section 6 sets out conclusions.

## 2. Background

Many studies have proposed the implementation of artificial neural networks and deep learning architectures for the development of automatic systems able to recognize the suspect of cardiac anomalies [40,41,42]. In the literature the detection of cardiac anomalies has been investigated analyzing both heart sounds acquired by digital stethoscopes and ECG signals from portable devices.

Meintjes et al. [43] implemented continuous wavelet transform (CWT) scalograms and convolutional neural networks for the correct classification of the fundamental heart sounds in recordings of normal and pathological heart sounds. They implemented a methodology in order to distinguish between the first and second heart sounds using CWT decomposition and convolutional neural network (CNN) features. Results show the high potential in the use of CWT and CNN in the analysis of heart sounds compared to support vector machine (SVM), and k-nearest neighbors (kNN) classifiers. In [44] authors propose the classification of heart sounds on short, unsegmented recordings and normalized spectral amplitude of 5 s duration phonocardiogram segments was determined by fast Fourier transform and wavelet entropy by wavelet analysis. Spectral amplitude and wavelet entropy features were then combined in a classification tree. They achieved accuracy comparable to other algorithms obtained without the complexity of segmentation. Redlarski et al. [17] presented a new heart sound classification technique combining linear predictive coding coefficients, used for feature extraction, with a classifier built upon combining support vector machine and the modified cuckoo search algorithm. It showed good performance of the diagnostic system, in terms of accuracy, complexity [45,46,47] and range of distinguishable heart sounds.

With the application of deep learning architectures, also the accuracy of ECG diagnostic analysis has achieved new high levels. The systems implemented using such techniques allow the automated interpretation of ECG signals from portable devices in real time. The common deep learning networks for the analysis of ECG signals are mainly based on recurrent neural networks (RNNs), convolutional neural networks (CNNs), and some other architectures [1].

Chauhan et al. [48] investigated the applicability of deep recurrent neural network architectures with long short term memory (LSTM) for detecting cardiac arrhythmias in ECG signals. This approach is quite fast, does not require preprocessing of the data [49] or hand coded features and do not need prior information about the abnormal signal. The network was tested on the MIT-BIH Arrhythmia Database for the classification of four different types of Arrhythmias showing that LSTMs may be a viable candidate for anomaly detection in ECG signals.

Saadatnejad et al. [50] proposed an LSTM-based ECG classification algorithm for continuous cardiac monitoring on wearable devices. They preprocessed data extracting RR interval and wavelet features from ECG samples. The ECG signal along with the extracted features were fed into multiple LSTM recurrent neural networks. The MIT-BIH ECG Arrhythmia Database was used for the classification of six different types of anomalies. The proposed algorithm achieved accurate LSTM-based ECG classification to wearable devices with low computational costs.

Thill et al. [51] presented an unsupervised time series anomaly detection algorithm to detect anomalies in ECG readings. They performed a recurrent LSTM network to predict the normal time series behavior without the usage of the anomaly class labels building a multivariate normal error model for the nominal data. Anomalous events were detected with a high probability through a high Mahalanobis distance. They classified six anomaly classes and obtained good performance achieving high levels of precision and recall.

Although RNN architectures are suitable to process time series data, they present some limitations. The major drawbacks of RNNs are the vanishing gradient and gradient exploding problems that make their training difficult, not allowing the processing of very long sequences. Moreover, due to its recurrent nature, the computation is slow. For this reason, some studies investigated the implementation of 1D-CNNs, with the main aim to design low computational complexity systems to support portable devices. In fact, Kiranyaz et al. [52] revised the state of the art techniques used in signal processing applications such as patient-specific ECG classification, structural health monitoring, anomaly detection in power electronics circuitry, and motor-fault detection. In particular, they highlighted how the implementation of adaptive and compact 1D-CNN can achieve higher performance than deep conventional 2D with low computational complexity. Adaptive and compact 1D-CNNs can be efficiently trained with a limited data set of 1D signals instead of massive size data sets required by deep 2D CNNs. It can be performed directly to the raw signal without any pre or post processing, such as features extraction, selection, dimensionality reduction, etc., and it is able to extract features from shorter segments of the overall data set. Moreover, due to the low computational requirements, 1D-CNNs are well suited for real-time and low-cost applications, especially on smart mobile devices that can be the proper tools for personal health monitoring [52,53]. Yıldırım et al. [54] proposed a new 1D-convolutional neural network approach for the automatic classification of cardiac arrhythmia on long-duration electrocardiography (ECG) signal analysis. The model was performed on 10-s fragments of ECG signals including 17 different classes of cardiac arrhythmia. The model showed remarkable performance and could be implemented with low computational complexity on mobile devices and cloud computing for tele-medicine, e.g., patient self-monitoring and preventive health. Li et al. [55] proposed a 1D-CNN based model to classify ECG signals. The model consisted of five layers and realized the classification of five typical kinds of arrhythmia signals. It achieved promising classification accuracy and significantly outperformed several typical ECG classification methods. Zubair et al. [56] propose an ECG beat classification system using a 1D-CNN model. The proposed classification system efficiently classified ECG beats into five different classes. Results showed that the model achieved a significant classification accuracy and superior computational efficiency than most of the state-of-the-art methods for ECG signal classification. Avanzato et al. [57] proposed a new neural architecture based on 1D-CNN for the development of automatic heart disease diagnosis systems using ECG signals. The model was performed on 30 s segments and classified three different classes of anomalies. It showed high performance and low complexity implementation. Kamaleswaran et al. [58] introduced a novel deep learning architecture for detection of normal sinus rhythm, AF, other abnormal rhythms, and noise. They proposed an optimal 13-layer 1D-CNN model with identified normal, AF and other rhythms using single lead short ECG recordings. The architecture was computationally fast and could also be used in real-time cardiac arrhythmia detection applications.

## 3. ECG Signal

The mechanical pumping activity of the heart muscle is determined by the rhythmic generation of an electrical impulse that originates at the level of the sinoatrial node and, through specialized conduction pathways, spreads to all cardiac muscle cells causing cycles of depolarization and repolarization underlying the contraction of single cells.

**Figure 1:**
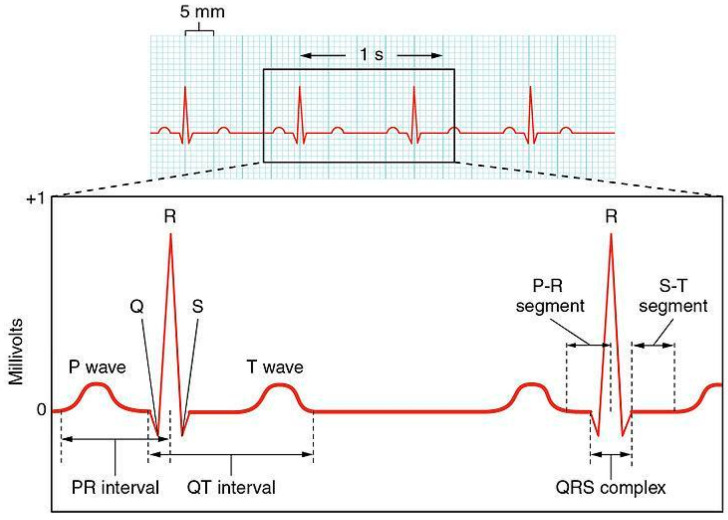
Tracing of a normal electrocardiogram (ECG) including P wave, QRS complex, and T wave. Reproduced with permission from Gordon Betts et al., Anatomy and Physiology, Connexions Website. http://cnx.org/content/col11496/1.6/; published by OpenStax, 19 June 2013.

ECG is the graphic reproduction of the electrical activity of the heart during its functioning, recorded at the surface of the body. The doctor, usually a cardiologist specialist, interprets the electrocardiographic recording by detecting the presence of cardiac arrhythmias, structural changes in the cardiac cavities, atria and/or ventricles, ischemia, myocardial infarction, and other cardiopathies, characterized by an alteration of electrical conduction. A beat of ECG signals can be observed by five characteristic waves—P, Q, R, S, and T [59], where each wave is related to a specific interval of the polarization–depolarization cycle. The characteristic of the normal ECG is that varies only in the presence of problems. The fundamental morphology of the ECG is given by three deflections (P, QRS, and T), which represent the formation and diffusion of the cardiac electrical impulse along the pathways of the conduction system (Figure 1).

## 4. Materials and Methods

### 4.1. Dataset

The proposed method was tested on the MIT-BIH Arrhythmia Database supplied by PhysioNet, a web resource for complex physiologic signals databases [60]. The MIT-BIH Arrhythmia Database contains 48 half-hour extracts of two-channel ambulatory ECG recordings, obtained from 47 subjects: 25 males aged between 32 and 89 years and 22 females aged between 23 and 89 years. As described by the authors in [60], the database consists of 23 recordings randomly selected by a set of 4000 24-h ambulatory ECG recordings collected from a mixed population of hospitalized (approximately 60%) and ambulatory (approximately 40%) patients at Beth Israel Hospital in Boston. The remaining 25 recordings were selected from the same set in order to include less common but clinically significant arrhythmias that would not be well represented in a small random sample. The recordings were digitized at 360 samples per second per channel with an 11-bit resolution over a 10-mV range. For our analysis, we used the same data published by Kaggle in txt and csv format, since they were easier to process [61].

The database contains 22 classes, 1 for normal beat, and 21 for various kinds of anomalies in ECG recordings.

### 4.2. Data Organization

Data were structured in a tabular form in order to be processed by the neural network. The ECGs are two-channel recordings including main derivations, varied among subjects. In most ECGs one channel is a Modified-Lead II (MLII) and the other channel is generally V1, sometimes V2, V4, or V5, depending on the subject. For this reason, since the MLII is almost present in every ECGs, we only considered this lead. Four ECG recordings, only containing leads V1 and V5, were excluded from the analysis. The 30-min ECG recordings were fragmented into segments of 15 s. Since the recordings were digitized at 360 samples per second, each segment consisted of 5400 samples (Table 1 and Table 2). Each segment was included in the analysis and no data cleaning process was executed.

Based on the annotations assigned to the peaks present, each segment was labeled as follows: if in the segment all peaks were annotated as normal then the entire segment was labeled as normal; if in the segment at least one peak was annotated as anomalous, presenting any kind of anomalies, then the entire segment was labeled as anomalous.

In the dataset, each row represented a segment of 15 s labeled as normal or anomalous. Labeled as normal were 2105 segments and 3175 as anomalous. Since the dataset presented clearly imbalanced classes showing a proportion bias, we undersampled the segments labeled as anomalous in order to keep only a part of these data, thus balancing the training set. The anomalous segments in excess were included in the test set.

**Figure 2:**
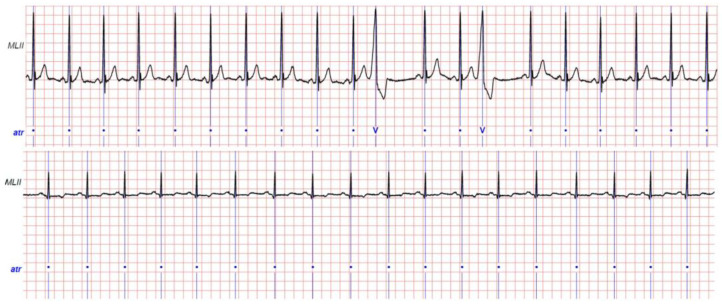
Examples of ECG recordings. (**Top**) Premature ventricular contraction (V). (**Bottom**) Normal record.

As will be detailed later, we carried out two distinct experiments, preprocessing data in different ways. In the first experimentation, segments were randomly included in the training set, using an equal proportion of segments for the two classes. The training set consisted of 2930 segments, 1465 labeled as normal, and 1465 labeled as anomalous. The training set was split in 70% for training and 30% for validation, keeping the same proportion of normal and anomalous segments. Segments contained in the test set presented a different proportion of the two classes, including 640 labeled as normal (60%) and 426 as anomalous (40%). Examples of normal and anomalous ECG recording are shown in Figure 2. Table 3 shows the number of ECG segments for each normal or anomalous class. The segments classified as anomalous can include one or more types of anomalies.

In the first experiment, as a widespread procedure used in literature and in the studies we compared to our work, we randomly assigned segments extracted from the same ECG recordings into training and test set. In the second experiment, we carried out a patient-oriented analysis, assigning to the training set only segments extracted from the same ECG recordings. This procedure was carried out for ECGs from 28 patients, since each ECG is related to a single patient. Segments extracted from the remaining 16 ECGs were assigned to the test set. In this way, segments of the same patient ECG recording are not interleaved between the training and test set. The training set consisted of 3360 segments, 1594 normal, and 1766 anomalous and the test set included 1800 segments, 435 normal, and 1365 anomalous. With this selection procedure, every segment of ECG recordings included in the test set had never been presented to the network during the training phase. Thus, the evaluation is only done on ECG recordings of patients never seen by the model.

### 4.3. Model Architecture

Convolutional neural network is a special kind of artificial neural network developed for image classification in which the model normally processes two-dimensional spatial input data representing an image’s pixels, in a process called feature learning. The same model can be used for one-dimensional sequence of data, such as an analysis of time series data, signal data, or natural language processing. The architecture of the model is described in [62]. The electrocardiogram signal is a time series data sequence that represents electrical impulses from the myocardium [63]. Thus, we propose a 1D convolutional neural network consisting of four convolutional blocks and one output block for the analysis of ECG signal data, in order to automatically identify normal and anomalous ECG recordings.

**Figure 3:**
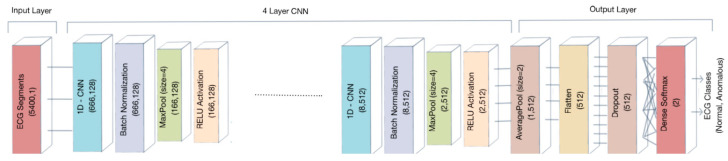
Architecture of the proposed 1D-convolutional neural network (CNN) model.

The convolutional block, as represented in Figure 3, consists of a 1D convolutional layer, a batch normalization layer, a 1D max pooling layer, and a rectified linear unit (ReLU) layer, while the output contains a 1D average pooling, a flatten layer, a dense layer, and a Softmax layer. We chose a four convolutional blocks architecture, since it showed a right tradeoff between computational efficiency and results accuracy.

The 1D convolutional layer creates a convolution kernel that is convolved with the input layer over a single dimension to produce a tensor of output. The kernel size was set to 80 in the first layer and decreased to 4 in the subsequent layers, in order to reduce computational costs (Table 4). The batch normalization standardized the input and it was applied after each convolutional layer and before the level of pooling, in order to improve performance and stabilize the learning process of the deep neural network [59]. The output of the batch normalization layer was downsampled by means of a 1D max pooling layer with a pool size of 4.

The 1D max pooling resizes the input representation by taking the maximum value on the window defined by pool size. The strides specify how much the pooling window moved for each pooling step. The pooling level was placed before ReLU to reduce overfitting.

In the output block, the 1D average pooling performed the same operation as the 1D max pooling but took the average window value. After the average pooling, the network had a flatten layer in order to transform multi-dimensional input feature vectors obtained in the previous layer to the appropriate size, as the input of the subsequent layers of the network. The output of the flatten layer is the input of a dense fully connected layer, which uses the Softmax function to predict output classes. Moreover, we used a dropout parameter to prevent overfitting. Dropout is a regularization technique that helps to reduce interdependent learning amongst the neurons. At each training, a set of neurons randomly chosen is dropped out of the net. We used dropout in the dense layer with a fraction of 0.6. With the same aim of preventing overfitting, in the dense layer, we also used a weight regularization approach. We used an L2 regularization penalty (sum of the squared weights) with hyperparameter equal to 0.001 in order to keep small values of weight in the dense layer. The architecture of the proposed 1D-CNN model is shown in Figure 3 and Table 4.

### 4.4. Validation and Performance Metrics

In order to evaluate the performance of our model, the training set was split in 70% for training and 30% for validation. Then to validate the stability of the model and generalize results, a resampling procedure was performed on the training set. In particular, we implemented k-fold cross-validation with k = 10. Data were split into 10 groups, then each group at a time was used as a validation set and the remaining groups as the training set. Data were split such that no observation could be included both in training and in test sets. The network was fitted on the training set and evaluated on the validation set for 10 times. Results were summarized with mean and standard deviation values of the model performance metrics. Finally, the testing set was used to verify the robustness of the neural network on data not included in the training set.

To evaluate the performance of the neural network, we computed the confusion matrix and the traditional classification metrics. In particular, given TP (true positive) and TN (true negative) the number of events correctly classified, respectively, as successes or failures and FP (false positive) and FN (false negative) the number of events incorrectly classified, respectively, as successes or failures, we calculated

(1) $$\mathrm{Accuracy}=\frac{\mathrm{TP}+\mathrm{TN}}{\mathrm{TP}+\mathrm{TN}+\mathrm{FN}+\mathrm{FP}},$$

(2) $$\mathrm{Precision}=\frac{\mathrm{TP}}{\mathrm{TP}+\mathrm{FP}},$$

(3) $$\mathrm{Recall}=\frac{\mathrm{TP}}{\mathrm{TP}+\mathrm{FN}},$$

(4) $$F1=\frac{2\mathrm{TP}}{2\mathrm{TP}+\mathrm{FP}+\mathrm{FN}},$$

## 5. Results and Discussion

We performed the proposed 1D convolution neural network on the MIT-BIH Arrhythmia data processed as segments of 15 s consisting of 5400 samples including 21 different classes of anomalies. 

**Figure 4:**
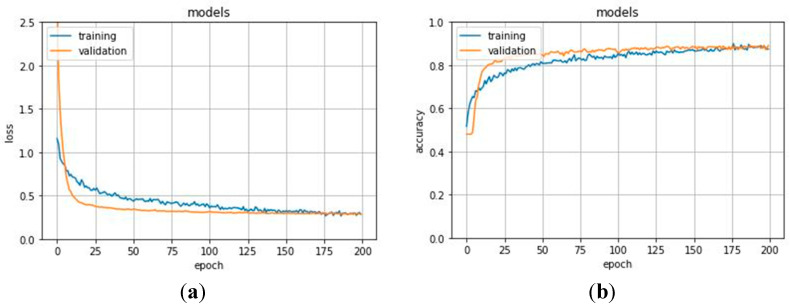
(**a**) Training and validation loss and (**b**) training and validation accuracy for the first experimentation. Accuracy metric is defined in Equation (1). Epoch is commonly referred to as the number of a full training pass over the entire dataset.

In the first experiment, we split the dataset basing on segments as explained in Section 4.2. The network was trained on 70% of the set and was validated on the remaining 30% for 200 epochs. Results for the validation performed on the training set are shown in Figure 4. The network stabilized in convergence in a training process of 200 epochs. The learning curves of the training and validation loss stabilized below 0.5 and the learning curves of the training and validation accuracy stabilized around 90%, both with a minimal gap between the final values.

In order to validate the stability of the proposed method, we used a k-fold cross validation as explained in Section 4.4. The average accuracy of the model was 89.3 ± 0.26% of standard deviation and the average loss was of 0.28 ± 0.06% and an average recall 85.6 ± 0.03%.

In order to assess the performance, the network was evaluated on the test set. The confusion matrix and the related metrics were computed (Table 5). The network showed an accuracy of 89.51%, and a recall of 91.09% for normal and 87.79% for anomalous segments, the precision of 91.81% for normal and 86.78% for anomalous segments, and F1-score 91.45% for normal and 87.28% for anomalous segments.

We compared the proposed network with the studies that, at the state of the art, performed the same 1D convolutional neural network using the MIT-BIH Arrhythmia Dataset with the aim to implement an automatic classification of cardiac pathologies based on ECG signals. The results of this comparison are reported in Table 6.

Briefly summarizing, Yıldırım et al. [54] proposed a 1D-convolutional neural network model for cardiac arrhythmia (17 classes) detection based on long-duration electrocardiography (ECG) signal analysis. They designed a complete end-to-end structure with neither hand-crafted feature extraction of the signals nor feature selection at any stage using a 16-layer deep network structure including standard CNN layers. The network was tested on MIT-BIH Arrhythmia database considering 10 s ECG signal segments for one lead (MLII) for a total of 1000 ECG signal segments from 45 persons. The network achieved a detection accuracy of 17 cardiac arrhythmia disorders (classes) at a level of 91.33%. Li et al. [55] proposed a 1D-convolutional neural network to classify ECG signals. The network consisted of five layers in addition to the input layer and the output layer, in particular, two convolution layers, two down sampling layers and one full connection layer. The model extracted the effective features from data and classified the features automatically. The wavelet threshold method was used to filter the high frequency noise, while the wavelet transform and reconstruction algorithm to correct the baseline drift, which is a low-frequency noise. Subsequently, the segmentation of ECG signals and the reduction of dimensions were performed by using R peaks that were located by the method of the wavelet transform. The network achieved a detection accuracy of five typical kinds of arrhythmia signals at a level of 97.5%. Zubair et al. [56] proposed an ECG beat classification system based on a 1D-convolutional neural network. The model integrated feature extraction and classification of ECG pattern recognition and consisted of three convolution layers, three pooling layers, a multilayer perceptron and Softmax layer. The classification was performed in three main steps: ECG beat detection, sample extraction, and classification. ECG beat detection stage involves the detection of the individual beat signal from 30 min long ECG recording of each patient, using modified-Lead II signals. Equal numbers of samples (100) on both right and left sides from the Rpeak were extracted and downsampling was performed to represent raw data of each beat by 128 samples. The network classified five different kinds of anomalies with a detection accuracy of 92.7%. Avanzato et al. [57] proposed an automatic heart disease diagnosis system using ECG signals based on the direct application of a 1D-convolutional neural network. The network consisted of three layers in addition to the input layer and the output layer. Unlike the classic CNN, which use fully connected neurons as their output layer, this network performed a single Average pooling layer and then a Softmax followed by a natural logarithm. The structure of the neural network input consisted of 30-s segments where every second of ECG recording was equivalent to 360 samples, for a total of 10,800 samples. The paper evaluated the performance of the network on three classes of anomalies with an accuracy of 98.33%.

Table 6 shows that the proposed method presents remarkable performance compared to the other studies, considering that it is able to detect anomalies in an ECG recording including 21 different classes. In addition, the proposed method is able to classify anomalies even in presence of more than one kind of cardiac pathology in the same ECG segment. It achieves recall and F1-score, respectively of 87.79% and 86.78%, improving results obtained in [54], which considered only 17 classes of anomalies. These results were achieved with a detection accuracy of 89.51%, slightly lower than the accuracy obtained using the method proposed in [54], despite the increase in the number of different kinds of anomalies. The high performance obtained for recall, in spite of a small loss for accuracy, is consistent with the research goal to reduce the number of ECGs sent to the medical specialist for further examination. At the same time, we need to ensure that anomalous ECGs could not escape the medical examination and the prescription of the proper therapy. Although results obtained in [54,55,56] showed higher performance, in those studies the analysis was carried out including a lower number of anomalies. In particular, [55,56] included in the analysis five classes and obtained an accuracy, respectively, of 97.5% and 92.7%. In [57] the analysis was conducted using three classes with an accuracy of 98.33%. Note that the model in [54] was based on 16 levels architecture (therefore with a large computational complexity to deploy, as an example, on wearable devices) and the models in [55,56,57], although based on four levels, operate only on a very small number of anomalies.

Analyzing the 52 segments containing anomalies and incorrectly classified as normal (false negative, see Table 5), we observed that, for most of them, at least one of the segments extracted from the same ECG recording was correctly classified as anomalous. Overall, only five ECG recordings from patients affected by cardiac pathologies were not detected at all. 

In light of this, we carried out a second experiment, including in the training set only segments extracted from the same ECG recordings, and for the test set, as explained in Section 4.2. Thus, the proposed 1D-CNN was tested on segments extracted from ECG recordings never presented to the network in the training process.

**Figure 5:**
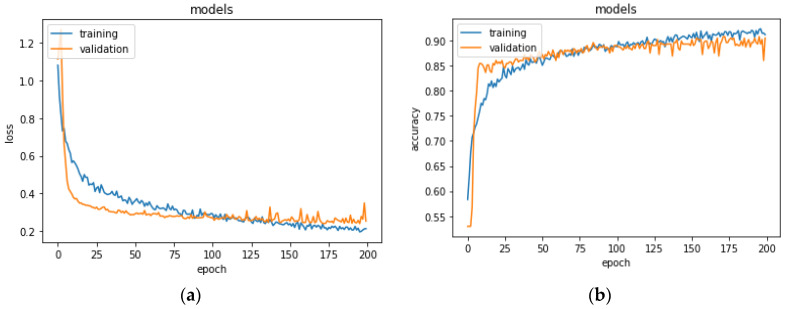
(**a**) Training and validation loss and (**b**) training and validation accuracy for the second experimentation.

The training set consisted of 3360 segments, 1594 normal, and 1766 anomalous, extracted from 28 ECG recordings, whereas the remaining recordings were included in the test set. In the test set, there were 1800 segments, 435 normal, and 1365 anomalous. To evaluate the performance of the model, the network was trained on 70% of the set and was validated on the remaining 30% for 200 epochs. Figure 5 shows the learning process for training and validation loss and accuracy. The model stabilized in convergence in a training process of 200 epochs. Additionally, in this case, the learning curves of the training and validation loss stabilized around zero and the learning curves of the training and validation accuracy stabilized around 90%.

In order to validate the stability of the proposed method, we used a k-fold cross validation as explained in Section 4.4. The average accuracy of the model was 88.2 ± 0.28% of standard deviation and the average loss was of 0.29 ± 0.07% and an average recall of 87.6 ± 0.03%.

The network was, then, evaluated on the test set. The resulting confusion matrix and the related metrics are shown in Table 7.

The network showed an accuracy of 84.94%, and a recall of 55.40% for normal and 94.36% for anomalous segments, the precision of 75.79% for normal and 86.91% for anomalous segments, and F1-score 64.01% for normal and 90.48% for anomalous segments.

The model showed a higher recall for anomalous segments compared to the previous test. Analyzing the 77 segments containing anomalies and incorrectly classified as normal, we observed that, for all of them, at least one of the segments extracted from the same ECG recording was correctly classified as anomalous. This result showed a remarkable improvement in the performance of the proposed method since, thinking in terms of patients rather than segments, the system was able to detect 100% of ECG recordings from patients affected by cardiac pathologies.

Considering this performance, we believe that the unavoidable goal to detect anomalous segments and minimize missed alarms was reached. This claim must be considered in the operative context where the model was deployed, in which the cost of a false alarm was considerably less than a missed one.

Moreover, we want to highlight that these results were achieved with the second experiment whose settings represents a closer scenario in terms of model usage. In the second experiment the model was validated on ECG records of different patients never presented to the model during the training. This experiment design is not common since validation is usually carried out with the usual holdout method, that is, segments from the same ECG recordings could be interleaved between training and test sets. With holdout there are chances that some signal patterns have been already presented to the model in the training phase.

However, it should be noted that the system described here still presents a significant false alarm rate. We are currently investigating different strategies to reduce the required workload due to false alarms. One viable solution is to isolate and collect only anomalous detected segments; when they occur in significant quantities can be sent remotely for the expert verification. Looking only at some of the segments, just those classified as anomalous by our model, can be a very quick job for an expert, certainly less demanding than observing an entire ECG, even more so if referred to a Holter exam.

This operational solution is well suited also for continual and active learning techniques, that is to periodically retrain the model based on new annotations from an expert; in this way, as new examples come into the system a probable gradual decrease in false alarms events is expected, with more balanced performances.

## 6. Conclusions

The diffusion of personal portable monitoring devices could involve the reporting of millions of ECG recordings every day. Systems able to support the cardiologists’ work in the interpretation of ECGs for the diagnosis of cardiac pathologies are required to facilitate health care decision making reducing considerably the expenditure of time and money. In fact, the automated detection of suspicious anomalies in ECG recordings can drastically reduce the number of ECGs that need to be manually examined by the cardiologists, excluding those classified as normal.

In the present paper, we propose a system able to automatically detect the suspect of cardiac pathologies in ECG signals from personal monitoring devices, using a 1D-CNN architecture. The 1D-CNN model overcomes the problems of vanishing gradient and gradient exploding related to recurrent neural networks, making their training difficult. Moreover, the 1D-CNN model allows one to implement real-time and low-cost systems and it is characterized by low computational complexity, feasible implementation on smart devices, and cloud computing. The system was optimized to detect the suspect of anomalies classifying normal and anomalous ECG, regardless of the kind of cardiac pathology. The proposed model was tested on the MIT-BIH ECG Arrhythmia Database, which included 21 different classes of ECG anomalies. Two different experimentations were carried out showing remarkable performance compared to the other studies conducted using the 1D-CNN architecture tested on the MIT-BIH ECG Arrhythmia Database. In particular, the network achieved accuracy and recall, respectively, of 84.94% and 94.36% computed with respect to the ECG signal segments and accuracy and recall of 100% when computed with respect to the patients, therefore considering the detection of anomalies in the entire ECG recordings.

We are now working on a possible personalization of the model, tunable toward a single person. We expect that the performance of this kind of model could be much better than a general one, with an additional cost of model calibration before the actual usage.

In the same study we are also investigating a model trained on “normal” segments of healthy patients and abnormal segments of pathological patients (since it is not possible to obtain abnormal segments from a healthy patient). This will allow us to observe if the model trained on this new dataset presents different characteristics than the model described in this paper. Here, we wanted to compare our study with studies that were as homogeneous as possible, at least in the dataset used, so we did not introduce any further changes regarding the training data.

## Figures and Tables

**Table 1:** Example of one 30-min ECG recording digitized at 360 samples per second provided by Kaggle. (**a**) Each row is a sample of Modified-Lead II MLII and V5 lead signals quantized with 11-bit resolution over a ±5 mV range. Sample values thus range from 0 to 2047 inclusive, with a value of 1024 corresponding to zero volts; (**b**) only includes annotated samples f(N = normal or A = anomalous) with a timestamp.

Sample	MLII	V5	Time	Sample	Type
0	995	1011	0:00.214	77	N
1	1000	1008	0:01.028	370	N
2	997	1008	0:01.839	662	N
…	…	…	…	…	…
648,000	969	997	30:00.564	648,203	A
648,000	969	1003	30:01.325	648,477	N
(**a**)			(**b**)		

**Table 2:** Data organization of one 30-min ECG recording fragmented into segments. Columns from MLII_0 to MLII_539 represent samples contained in 15 s fragment. Each row is a different and consecutive fragment. Type N = normal, A = anomaly.

MLII_0	MLII_1	MLII_2	MLII_3	…	MLII_5396	MLII_5397	MLII_5398	MLII_5399	Type
995	1000	997	995	…	977	979	975	974	N
…	…	…	…	…	…	…	…	…	…
989	988	986	990	…	974	972	969	969	A

**Table 3:** ECG segments for each ECG class in train and test sets for the first experimentation. Since our goal is to detect any kind of anomaly, we considered both rhythm and beat alterations with no difference. We also considered signal related annotations since a low quality noise signal could represent an issue to medical diagnosis.

Type	Class	Train	Test
Normal	Normal beat	1465	640
Anomalous	Left bundle branch block beat	331	123
	Right bundle branch block beat	330	131
	Atrial premature beat	406	150
	Aberrated atrial premature beat	49	29
	Nodal (junctional) premature beat	5	2
	Supraventricular premature beat	1	0
	Premature ventricular contraction	1236	520
	Fusion of ventricular and normal beat	165	74
	Ventricular flutter wave	12	2
	Atrial escape beat	12	4
	Nodal (junctional) escape beat	45	14
	Ventricular escape beat	5	4
	Paced beat	151	86
	Fusion of paced and normal beat	46	30
	Non-conducted P-wave (blocked APB)	26	16
	Unclassifiable beat	6	4
	Isolated QRS-like artifact	55	30
	Change in signal quality (qq)	253	115
	Rhythm change	432	189
	Start of ventricular flutter/fibrillation	5	1
	End of ventricular flutter/fibrillation	5	1

**Table 4:** Detailed parameters of the proposed 1D-CNN model.

Input Shape (5400;1)–Sample of 5400 Samples		
Type of Layer	Output Shape	Other Parameters
Conv1D	(666;128)	Kernel size = 80, Strides = 8, Filters = 128
Batch Normalization	(666;128)	-
Max Pooling 1D	(166;128)	Pool size = 4
Activation	(166;128)	ReLU activation
Conv1D	(163;256)	Kernel size = 4, Filters = 256
Batch Normalization	(163;256)	-
Max Pooling 1D	(40;256)	Pool size = 4
Activation	(40;256)	ReLU activation
Conv1D	(37;256)	Kernel size = 4, Filters = 256
Batch Normalization	(37;256)	-
Max Pooling 1D	(9;256)	Pool size = 4
Activation	(9;256)	ReLU activation
Conv1D	(8;512)	Kernel size = 4, Filters = 512
Batch Normalization	(8;512)	-
Max Pooling 1D	(2;512)	Pool size = 4
Activation	(2;512)	ReLU activation
AveragePooling 1D	(1;512)	Pool size = 2
Flatten	(512)	-
Dropout	(512)	Rate 0.6
Dense	(2)	Regularizer L2 (0.001), Softmax activation

**Table 5:** Confusion matrix and related performance metrics for the test set in the first experimentation.

		Predicted		Accuracy	Recall	Precision	F1
		Normal	Anomalous	(%)	(%)	(%)	(%)
True	Normal	583	57	89.51	91.09	91.81	91.45
	Anomalous	52	374		87.79	86.78	87.28

**Table 6:** Comparison with studies performed using 1D-CNN on the MIT-BIH Arrhythmia Database.

Article	Model	Classes of Anomalies	Accuracy	Recall	F1-Score	
[54]	1D-CNN	17 classes	91.3%	83.9%		85.4%
[55]	1D-CNN	5 classes	97.5%	-		-
[56]	1D-CNN	5 classes	92.7%	-		-
[57]	1D-CNN	3 classes	98.33%	98.33%		98.33%
Proposed method	1D-CNN	1 class, including 21 kinds of anomalies	89.51%	87.79%		86.78%

**Table 7:** Confusion matrix and related performance metrics for the test set in the second experimentation.

		Predicted		Accuracy	Recall	Precision	F1
		Normal	Anomalous	(%)	(%)	(%)	(%)
True	Normal	241	194	84.94	55.40	75.79	64.01
	Anomalous	77	1288		94.36	86.91	90.48

## Data Availability

The proposed method was tested on the MIT-BIH Arrhythmia Database supplied by PhysioNet, see https://www.physionet.org/content/mitdb/1.0.0/.

## References

[1] Cai W., Hu D., Liu C., Li J. (2020). ECG Interpretation with Deep Learning. Feature Engineering and Computational Intelligence in ECG Monitoring.

[2] Chamley R.R., Holdsworth D.A., Rajappan K., Nicol E.D. (2019). ECG interpretation: Interpretation of the ECG in young, fit, asymptomatic individuals undertaking high-hazard occupations is the topic of the fourth article in the occupational cardiology series. Eur. Heart J..

[3] Dimauro G., Caivano D., Ciccone M.M., Dalena G., Girardi F. (2021). Classification of cardiac tones of mechanical and native mitral valves. Ambient Assisted Living, Lecture Notes in Electrical Engineering.

[4] Schläpfer J., Wellens H.J. (2017). Computer-Interpreted Electrocardiograms. J. Am. Coll. Cardiol..

[5] Dimauro G., de Ruvo S., di Terlizzi F., Ruggieri A., Volpe V., Colizzi L., Girardi F. (2020). Estimate of Anemia with New Non-Invasive Systems—A Moment of Reflection. Electronics.

[6] Dimauro G., Bevilacqua V., Colizzi L., di Pierro D. (2020). TestGraphia, a Software System for the Early Diagnosis of Dysgraphia. IEEE Access.

[7] Dimauro G., Guarini A., Caivano D., Girardi F., Pasciolla C., Iacobazzi A. (2019). Detecting Clinical Signs of Anaemia From Digital Images of the Palpebral Conjunctiva. IEEE Access.

[8] Kasiviswanathan S., Vijayan T.B., Simone L., Dimauro G. (2020). Semantic Segmentation of Conjunctiva Region for Non-Invasive Anemia Detection Applications. Electronics.

[9] Monaco A., Cattaneo R., Mesin L., Fiorucci E., Pietropaoli D. (2014). Evaluation of autonomic nervous system in sleep apnea patients using pupillometry under occlusal stress: A pilot study. Cranio.

[10] Castroflorio T., Mesin L., Tartaglia G.M., Sforza C., Farina D. (2013). Use of electromyographic and electrocardiographic signals to detect sleep bruxism episodes in a natural environment. IEEE J. Biomed. Health Inform..

[11] Bevilacqua V., Pietroleonardo N., Triggiani V., Brunetti A., Di Palma A.M., Rossini M., Gesualdo L. (2017). An innovative neural network framework to classify blood vessels and tubules based on Haralick features evaluated in histological images of kidney biopsy. Neurocomputing.

[12] Buongiorno D., Cascarano G.D., De Feudis I., Brunetti A., Carnimeo L., Dimauro G., Bevilacqua V. (2020). Deep Learning for Processing Electromyographic Signals: A Taxonomy-based Survey. Neurocomputing.

[13] Estes N.A.M. (2013). Computerized Interpretation of ECGs: Supplement Not a Substitute. Circ. Arrhythm. Electrophysiol..

[14] Kligfield P., Gettes L.S., Bailey J.J., Childers R., Deal B.J., Hancock E.W., van Herpen G., Kors J.A., Macfarlane P., Mirvis D.M. (2007). Recommendations for the Standardization and Interpretation of the Electrocardiogram: Part II: The Electrocardiogram and Its Technology: A Scientific Statement From the American Heart Association Electrocardiography and Arrhythmias Committee, Council on Clinical Cardiology; the American College of Cardiology Foundation; and the Heart Rhythm Society: Endorsed by the International Society for Computerized Electrocardiology. Circulation.

[15] Fye W.B. (1994). A History of the origin, evolution, and impact of electrocardiography. Am. J. Cardiol..

[16] Mesin L., Aram S., Pasero E. (2014). A neural data-driven algorithm for smart sampling in wireless sensor networks. J. Wirel. Commun. Netw..

[17] Redlarski G., Gradolewski D., Palkowski A. (2014). A System for Heart Sounds Classification. PLoS ONE.

[18] Liu W., Wang Z., Liu X., Zeng N., Liu Y., Alsaadi F.E. (2017). A survey of deep neural network architectures and their applications. Neurocomputing.

[19] Zhao W., Du S. (2016). Spectral–Spatial Feature Extraction for Hyperspectral Image Classification: A Dimension Reduction and Deep Learning Approach. IEEE Trans. Geosci. Remote Sens..

[20] Dimauro G., Deperte F., Maglietta R., Bove M., la Gioia F., Renò V., Simone L., Gelardi M. (2020). A Novel Approach for Biofilm Detection Based on a Convolutional Neural Network. Electronics.

[21] Renò V., Sciancalepore M., Dimauro G., Maglietta R., Cassano M., Gelardi M. (2020). A Novel Approach for the Automatic Estimation of the Ciliated Cell Beating Frequency. Electronics.

[22] Dimauro G., Bevilacqua V., Fina P., Buongiorno D., Brunetti A., Latrofa S., Cassano M., Gelardi M. (2020). Comparative Analysis of Rhino-Cytological Specimens with Image Analysis and Deep Learning Techniques. Electronics.

[23] Dimauro G., Altomare N., Scalera M. PQMET: A digital image quality metric based on human visual system. Proceedings of the 2014 4th International Conference on Image Processing Theory, Tools and Applications (IPTA).

[24] Dimauro G., Simone L. (2020). Novel Biased Normalized Cuts Approach for the Automatic Segmentation of the Conjunctiva. Electronics.

[25] Sainath T.N., Weiss R.J., Wilson K.W., Li B., Narayanan A., Variani E., Bacchiani M., Shafran I., Senior A., Chin K. (2017). Multichannel Signal Processing With Deep Neural Networks for Automatic Speech Recognition. IEEE/ACM Trans. Audio Speech Lang. Process..

[26] Barletta V.S., Caivano D., Nannavecchia A., Scalera M. (2020). Intrusion Detection for in-Vehicle Communication Networks: An Unsupervised Kohonen SOM Approach. Future Internet.

[27] Barletta V.S., Caivano D., Nannavecchia A., Scalera M. (2020). A Kohonen SOM Architecture for Intrusion Detection on In-Vehicle Communication Networks. Appl. Sci..

[28] Barletta V.S., Caivano D., Dimauro G., Nannavecchia A., Scalera M. (2020). Managing a Smart City Integrated Model through Smart Program Management. Appl. Sci..

[29] Renò V., Dimauro G., Labate G., Stella E., Fanizza C., Cipriano G., Carlucci R., Maglietta R. (2019). A SIFT-based software system for the photo-identification of the Risso’s dolphin. Ecol. Inform..

[30] Dimauro G., Colagrande P., Carlucci R., Ventura M., Bevilacqua V., Caivano D. (2019). CRISPRLearner: A Deep Learning-Based System to Predict CRISPR/Cas9 sgRNA On-Target Cleavage Efficiency. Electronics.

[31] Ribeiro A.H., Ribeiro M.H., Paixão G.M.M., Oliveira D.M., Gomes P.R., Canazart J.A., Ferreira M.P.S., Andersson C.R., Macfarlane P.W., Meira W. (2020). Automatic diagnosis of the 12-lead ECG using a deep neural network. Nat. Commun..

[32] Stead W.W. (2018). Clinical Implications and Challenges of Artificial Intelligence and Deep Learning. JAMA.

[33] Naylor C.D. (2018). On the Prospects for a (Deep) Learning Health Care System. JAMA.

[34] Dimauro G., Girardi F., Caivano D., Colizzi L., Leone A., Caroppo A., Rescio G., Diraco G., Siciliano P. (2019). Personal Health E-Record—Toward an Enabling Ambient Assisted Living Technology for Communication and Information Sharing Between Patients and Care Providers. Ambient Assisted Living.

[35] Ardito C., Caivano D., Colizzi L., Dimauro G., Verardi L. (2020). Design and Execution of Integrated Clinical Pathway: A Simplified Meta-Model and Associated Methodology. Information.

[36] Dimauro G., di Pierro D., Deperte F., Simone L., Fina P.R. (2020). A Smartphone-Based Cell Segmentation to Support Nasal Cytology. Appl. Sci..

[37] AliveCor. https://www.alivecor.com/kardiamobile/.

[38] D-Heart Smartphone ECG Device. https://www.d-heartcare.com/.

[39] eKuore | Wireless Electronic Stethoscope. https://www.ekuore.com/.

[40] Wang H., Shi H., Chen X., Zhao L., Huang Y., Liu C. (2020). An Improved Convolutional Neural Network Based Approach for Automated Heartbeat Classification. J. Med. Syst..

[41] Duan L., Hongxin Z., Zhiqing L., Juxiang H., Tian W. (2019). Deep residual convolutional neural network for recognition of electrocardiogram signal arrhythmias. J. Biomed. Eng..

[42] Wan X., Jin Z., Wu H., Liu J., Zhu B., Xie H. (2020). Heartbeat classification algorithm based on one-dimensional convolution neural network. J. Mech. Med. Biol..

[43] Meintjes A., Lowe A., Legget M. Fundamental Heart Sound Classification using the Continuous Wavelet Transform and Convolutional Neural Networks. Proceedings of the 2018 40th Annual International Conference of the IEEE Engineering in Medicine and Biology Society (EMBC).

[44] Langley P., Murray A. (2017). Heart sound classification from unsegmented phonocardiograms. Physiol. Meas..

[45] He S., Fataf N.A.A., Banerjee S., Sun K. (2019). Complexity in the muscular blood vessel model with variable fractional derivative and external disturbances. Phys. A Stat. Mech. Appl..

[46] He S., Sun K., Wang H. (2015). Complexity Analysis and DSP Implementation of the Fractional-Order Lorenz Hyperchaotic System. Entropy.

[47] He S., Sun K., Banerjee S. (2016). Dynamical properties and complexity in fractional-order diffusionless Lorenz system. Eur. Phys. J. Plus.

[48] Chauhan S., Vig L. Anomaly detection in ECG time signals via deep long short-term memory networks. Proceedings of the 2015 IEEE International Conference on Data Science and Advanced Analytics (DSAA).

[49] Scalera M., Antonella S. (2014). Customer centric strategies for value creation: Academic experimentation. J. E-Learn. Knowl. Soc..

[50] Saadatnejad S., Oveisi M., Hashemi M. (2020). LSTM-Based ECG Classification for Continuous Monitoring on Personal Wearable Devices. IEEE J. Biomed. Health Inf..

[51] Thill M., Däubener S., Konen W., Bäck T. Anomaly Detection in Electrocardiogram Readings with Stacked LSTM. Proceedings of the ITAT 2019 Information Technologies—Applications and Theory.

[52] Kiranyaz S., Ince T., Abdeljaber O., Avci O., Gabbouj M. 1-D Convolutional Neural Networks for Signal Processing Applications. Proceedings of the 2019 IEEE International Conference on Acoustics, Speech and Signal Processing (ICASSP).

[53] Kiranyaz S., Ince T., Abdeljaber O., Gabbouj M. (2016). Real-Time Patient-Specific ECG Classification by 1-D Convolutional Neural Networks. IEEE Trans. Biomed. Eng..

[54] Yıldırım Ö., Pławiak P., Tan R.-S., Acharya U.R. (2018). Arrhythmia detection using deep convolutional neural network with long duration ECG signals. Comput. Biol. Med..

[55] Li D., Zhang J., Zhang Q., Wei X. Classification of ECG signals based on 1D convolution neural network. Proceedings of the 2017 IEEE 19th International Conference on e-Health Networking, Applications and Services (Healthcom).

[56] Zubair M., Kim J., Yoon C. An Automated ECG Beat Classification System Using Convolutional Neural Networks. Proceedings of the 2016 6th International Conference on IT Convergence and Security (ICITCS).

[57] Avanzato R., Beritelli F. (2020). Automatic ECG Diagnosis Using Convolutional Neural Network. Electronics.

[58] Kamaleswaran R., Mahajan R., Akbilgic O. (2018). A robust deep convolutional neural network for the classification of abnormal cardiac rhythm using single lead electrocardiograms of variable length. Physiol. Meas..

[59] Hsieh C.-H., Li Y.-S., Hwang B.-J., Hsiao C.-H. (2020). Detection of Atrial Fibrillation Using 1D Convolutional Neural Network. Sensors.

[60] Moody G.B., Mark R.G. (2001). The impact of the MIT-BIH arrhythmia database. IEEE Eng. Med. Biol. Mag..

[61] Mondejar V. Kaggle MIT-BIH Arrhythmia Database. https://www.kaggle.com/mondejar/mitbih-database.

[62] Wang C., Xi Y. (1997). Convolutional Neural Network for Image Classification.

[63] Sivaraks H., Ratanamahatana C.A. (2015). Robust and Accurate Anomaly Detection in ECG Artifacts Using Time Series Motif Discovery. Comput. Math. Methods Med..

